# Airway Redox Homeostasis and Inflammation Gone Awry: From Molecular Pathogenesis to Emerging Therapeutics in Respiratory Pathology

**DOI:** 10.3390/ijms21239317

**Published:** 2020-12-07

**Authors:** Javier Checa, Josep M. Aran

**Affiliations:** Immune-Inflammatory Processes and Gene Therapeutics Group, IDIBELL, L’Hospitalet de Llobregat, 08908 Barcelona, Spain; jcheca@idibell.cat

**Keywords:** oxidative stress, inflammation, respiratory diseases, therapeutic strategies

## Abstract

As aerobic organisms, we are continuously and throughout our lifetime subjected to an oxidizing atmosphere and, most often, to environmental threats. The lung is the internal organ most highly exposed to this milieu. Therefore, it has evolved to confront both oxidative stress induced by reactive oxygen species (ROS) and a variety of pollutants, pathogens, and allergens that promote inflammation and can harm the airways to different degrees. Indeed, an excess of ROS, generated intrinsically or from external sources, can imprint direct damage to key structural cell components (nucleic acids, sugars, lipids, and proteins) and indirectly perturb ROS-mediated signaling in lung epithelia, impairing its homeostasis. These early events complemented with efficient recognition of pathogen- or damage-associated recognition patterns by the airway resident cells alert the immune system, which mounts an inflammatory response to remove the hazards, including collateral dead cells and cellular debris, in an attempt to return to homeostatic conditions. Thus, any major or chronic dysregulation of the redox balance, the air–liquid interface, or defects in epithelial proteins impairing mucociliary clearance or other defense systems may lead to airway damage. Here, we review our understanding of the key role of oxidative stress and inflammation in respiratory pathology, and extensively report current and future trends in antioxidant and anti-inflammatory treatments focusing on the following major acute and chronic lung diseases: acute lung injury/respiratory distress syndrome, asthma, chronic obstructive pulmonary disease, pulmonary fibrosis, and cystic fibrosis.

## 1. Introduction

Due to its continuously exposed surface to the external environment, the lung exhibits a formidable defense system constituted by a high number of interacting mechanisms [1,2]. First, anatomical retention features such as the nasopharyngeal barrier filter particles or microorganisms higher than 2–3 µm [3,4,5]. Secondly, there are systems to expel the external elements, i.e., the cough [6], and the mucociliary system [7]. Whether or not the external and potentially harmful particles overcome these mechanisms, the humoral factors come into play, including bactericidal and antiviral secretions (mucins, defensins, lactoferrin, complement factors, etc.) [8,9], and cellular factors of the innate [10] and adaptive immune system. These include the airway epithelial cells [11]; the phagocytic cells that, in turn, comprise polymorphonuclear (PMN) cells such as neutrophils (the most abundant immune cell type) [12] or eosinophils [13]; monocytes and macrophages [14,15]; natural killer cells (NKC) [16]; mastocytes [17]; and dendritic cells [18]. All these cells recognize pathogen-associated molecular patterns (PAMPs) such as lipopolysaccharide (LPS) through pattern recognition receptors (PRRs) [19], and Toll-like receptors (TLR) are the most studied [20]. Their stimulation triggers the activation of antimicrobial genes and inflammatory cytokines and chemokines, as well as the direct response against antigens [21], activating the adaptive immune system, namely B and T lymphocytes [22,23].

Nevertheless, when one or several components of this intricate network of pulmonary defense mechanisms fail, homeostasis is disrupted, and respiratory pathology ensues causing oxidative stress and inflammation. This review briefly underscores the molecular mechanisms behind environmental stress (airborne pollutants, pathogens, and allergens) causing pulmonary immune-inflammatory responses and focuses on current pharmacological options, emerging, and promising therapeutic approaches and new investigational treatments targeting the most common acute and chronic respiratory illnesses.

## 2. Reactive Oxygen Species Production in the Airways

Environmental pollutants such as ozone (O_3_) and nitrogen dioxide (NO_2_) react with several molecules at the respiratory surface and generate secondary reactive oxygen species (ROS) such as superoxide radicals (O_2_**^·^**^−^), hydrogen peroxide (H_2_O_2_), and hydroxyl radicals (OH**^·^**) [24,25]. Additionally, lung cells generate ROS as by-products of aerobic metabolism involving enzymatic reactions in the mitochondrial electron transport chain (e.g., through activity of amine oxidases, α-ketoglutarate dehydrogenase (α-KGDH), and pyruvate dehydrogenase (PDH), and activation of the p66shc adaptor protein) [26,27,28]. Furthermore, ROS can be produced in peroxisomes [29], or by cytochrome P450 enzymes, cyclooxygenases, and lipoxygenases [30]. Nitric oxidase synthases (NOS) expand the spectrum of ROS producing reactive nitrogen species (NO_2_ or ONOO^−^) [31]. ROS are also produced as mediators of biological functions, with a role in inflammatory processes involving epithelial and endothelial cells, alveolar macrophages, and granulocytes [32,33]. NADPH oxidases (NOX) enzymes are involved in both bacterial killing and regulation of inflammatory mediators [34]. Indeed, dual oxidases, DUOX1 and DUOX2, the major isoform of NOX, are expressed preferentially in the respiratory epithelium [35,36,37].

## 3. Respiratory Surface: Antioxidant Defenses

The air–liquid interface covering the developed airways is an environment subjected to continuous oxidative stress. Accordingly, the respiratory epithelium is exposed to endogenous and also to environmental ROS. Therefore, it expresses a variety of antioxidant enzymes. Superoxide dismutases such as SOD3 (an extracellular SOD, EC-SOD) [38,39,40], highly-expressed in the lung at the extracellular matrix and at the cell surfaces [41], generate H_2_O_2_ which is detoxified by other enzymes. Catalase, decomposes H_2_O_2_ into H_2_O and O_2_, predominantly within alveolar macrophages and type II epithelial cells [42,43]. Glutathione (GSH) peroxidase (GPX) catalyzes the reduction of H_2_O_2_ or other peroxides to glutathione disulfide (GSSG) and H_2_O, of which GPX1 is thought to be responsible for 95% of overall lung tissue GPX activity [44]. Peroxiredoxins (PRX), with all six mammalian family members expressed in different compartments within the lung [45], particularly PRX I, III, V, and VI in the bronchial epithelium, PRX V and VI in the alveolar epithelium, and PRX I and III in alveolar macrophages, decompose H_2_O_2_ and protect against oxidative stress [46,47,48]. Thioredoxin (TRX), whose main antioxidant role is related to its ability to regenerate oxidized forms of PRX [49,50], catalyzes the reduction of disulfide bonds, modulates signal transduction pathways, and has anti-inflammatory properties [51,52]. Finally, glutaredoxins (GRX) participate in the reduction of oxidative modifications involving GSH [53,54].

The following small non-enzymatic low-molecular-weight antioxidant molecules are highly relevant: ascorbic acid (vitamin C) [55], uric acid [56], GSH [57,58], and α-tocopherol (vitamin E) [59]. These non-enzymatic molecules are the most prominent antioxidants reacting with reactive oxidant gases such as O_3_ and NO_2_ [60,61,62,63] and with the secondary oxidants generated by them, which can increase the oxidative injury [64]. Furthermore, the enzymatic antioxidants complement the function of these small molecules. Nuclear factor erythroid 2-related factor (Nrf2) regulates the transcription of both antioxidant genes coding for many of the above-highlighted enzymes and phase II detoxification genes [65,66,67,68].

## 4. Inflammation and Oxidative Stress in Pulmonary Diseases

A variety of immune and non-immune cells are activated during an inflammatory process. Each cell type releases cytokines and mediators that modify the activities of other cells, inducing an inflammatory network that progresses and resolves towards healthy homeostatic, or pathological outcomes. The lung is a vital organ for gas exchange and is constantly exposed to harmful airborne pathogens. Therefore, an immediate and intense protective/defensive inflammatory action is required to eliminate the invaders as early as possible. Nevertheless, excessive inflammation can be life threatening [1]. Consequently, a delicate balance between inflammation and anti-inflammation is essential for lung homeostasis and for the prevention of chronic inflammation [69]. Among the main inflammatory mediators involved in the pathogenesis of respiratory diseases are biochemical mediators such as histamine, thrombin, complement anaphylatoxins, prostaglandins, nitric oxide (NO), and molecules induced by oxidative stress [70]. These compounds mediate cell signaling and enhance cytokine production, among other activities.

Thus, airborne toxicants stimulate local ROS production inducing protein oxidation, lipoxidation, glycation end products, and DNA damage, and leading to mitochondrial dysfunction, cell death, the recruitment of inflammatory cells (mainly macrophages and neutrophils), profibrotic changes or mucus hypersecretion. These oxidative stress-mediated cellular processes drive the development of key environmental respiratory diseases such as acute lung injury/respiratory distress syndrome, asthma, chronic obstructive pulmonary disease, and pulmonary fibrosis, and affect the progression of the most common hereditary disease affecting the lung, i.e., cystic fibrosis.

### 4.1. Acute Pulmonary Inflammation

#### Acute Lung Injury (ALI) and Acute Respiratory Distress Syndrome (ARDS)

Acute respiratory distress syndrome (ARDS) and its milder form acute lung injury (ALI) are critical pulmonary dysfunctions caused by heterogeneous pathologic factors [71] involving acute development of respiratory failure and severe hypoxemia [72,73,74], bilateral diffuse lung infiltrations, and impaired alveolar liquid clearance [75]. Lung injury leads to pulmonary vascular permeability, increased production of proinflammatory factors and enhanced expression of the adhesion molecules necessary for leukocyte recruitment and neutrophil migration across the endothelial layer [76,77]. Activated neutrophils secrete cytotoxic agents such as granular enzymes, proinflammatory cytokines, bioactive lipids, and along with epithelium and endothelium, excessive ROS [78], which upregulate the expression of proinflammatory cytokines and adhesion molecules amplifying the tissue damage and pulmonary edema [79,80]. Furthermore, a priority upon initiation of mechanical ventilation for ALI patients is providing them 100% FiO2 (fraction of inspired oxygen). Nevertheless, protecting against hypoxemia may predispose to oxidative stress, which may be further enhanced by decreased levels of GSH [81].

ARDS treatment has not experienced significant advances in the last 50 years. Early detection is the best approach to attenuate the development of ALI/ARDS. Therefore, several imaging techniques (ultrasound, CT scan, and positron emission tomography) are being assessed for the diagnosis of capillary permeability edema in early phase ARDS, indicative of damage to the alveolar-capillary membrane, given the poor sensitivity of chest X-rays [82,83,84,85,86]. There is no efficient therapy available, although mechanical lung ventilation has long been applied as a supportive approach for healing lung oxygenation, supplemented by anti-inflammatory drugs. Corticosteroid therapy was first indicated for ARDS by Ashbaugh and colleagues [73]. Indeed, despite its relevant side effects, low-dose and extended steroid treatment seems to improve pulmonary physiology in ARDS patients [87]. Other traditional treatments are fluid and hemodynamic management [88] to decrease pulmonary edema. The objective is to maintain the lowest intravascular volume.

### 4.2. Chronic Pulmonary Inflammation

#### 4.2.1. Asthma

Asthma is a chronic inflammatory disorder of the airways in which gene–environment interactions involving a variety of cell types play an important role [89,90], although its overall pathogenesis remains unknown. The inflammatory response in asthma consists of the recognition of allergen patterns by TLRs [91]. Most asthmatics have type 2 inflammation, related to the presence of type 2 cytokines (interleukins IL-4, IL-5, and IL-14) and a variety of inflammatory cells (eosinophils, mast cells, basophils, type 2 T helper (Th2) lymphocytes, immunoglobulin E (IgE)-producing plasma cells, epithelial cells, and macrophages) [92]. Myeloid dendritic cells process allergens and release chemokine (C-C motif) ligands CCL17 and CCL22 to attract Th2 cells to the injured region [93]. IgE molecules sensitize mast cells to release cysteinyl leukotrienes (CysLTs) and prostaglandin D2 (PGD2) [94]. Damaged epithelial cells release CCL11 to recruit eosinophils, which attract more proinflammatory mediators [95]. Eosinophils also produce pro-resolving lipid mediators (PD1) and these stimulate IL-10 production and macrophage phagocytosis, promoting the resolution of inflammation [96]. Damaged epithelial cells release stem cell factor (SCF) to activate myofibroblasts to repair damaged epithelial cells. Airway epithelial cells also play a relevant role regulating type 2 inflammation via cytokines (IL-25, IL-33, and thymic stromal lymphopoietin) [92].

Oxidative stress is important in asthma [97,98]. Steroid therapy attenuates H_2_O_2_ [99], oxidative stress markers [100] and NO levels [101]. An increase in ROS production is inversely correlated with forced expiratory volume in 1 s (FEV1) [102]. Therefore, inflammation in asthma results in increased oxidative stress in the airways [103], in alveolar macrophages, and in eosinophils and neutrophils [104], associated with damage to a wide range of biologic molecules such as lipids and proteins [105,106]. Numerous cytokines such as tumor necrosis factor alpha (TNF-α), heparin-binding epidermal growth factor (HB-EGF), fibroblast growth factor 2 (FGF2), angiotensin II (AII), serotonin, and thrombin are found in the lung during inflammation and activate oxidases that lead to increased ROS in cell culture [107]. ROS decrease β-adrenergic function in the lung [108] and, consequently, airway hyperresponsiveness is produced by increasing the vagal tone due to damage to oxidant-sensitive beta-adrenergic receptors [109]. ROS also sensitize airway muscles to acetylcholine-induced contraction [110,111] and produce bronchial hyperactivity [112], promote histamine release from mast cells, and increase mucus secretion from airway epithelial cells [113]. Furthermore, ROS prompt endothelial barrier dysfunction through tight junctions disruption, which increases the permeability to fluid, inflammatory cells, and mediators [114,115], decrease numbers and function of epithelial cilia [116], and stimulate mucin secretion [117]. In addition, H_2_O_2_ activates mitogen-activated kinases (MAPKs) in tracheal myocytes [118] and stimulates the tracheal smooth muscle to contract [119,120,121].

Regarding antioxidant defenses, despite increased levels of GSH in the airways, the ratio of oxidized to reduced GSH also increases [57]. Other antioxidants such as ascorbate and alpha-tocopherol decrease [122,123], and SOD activity, but not SOD quantity [39], is likewise diminished [124,125,126,127,128,129]. Catalase activity is suppressed [130,131]. GPX1 can also promote both proinflammatory cytokine production and neutrophilia in response to LPS challenge [132], and ovalbumin-induced allergic asthma. In fact, GPX1 regulates T helper cell proliferation and differentiation toward Th2 and Th17 phenotypes [133]. Likewise, GPX3 is elevated in asthmatic airways through oxidant-induced activation of activator protein 1 (AP-1) [134,135]. Increased extracellular levels of TRX have been reported as well [136,137], indicating increased oxidative stress [49]. Lung tissue expression of GRX-1 is increased in models of allergic asthma [138,139]. Several reports have shown that GRX-1 mediates proinflammatory signaling in LPS-mediated lung inflammation or during allergic airways disease, possibly by regulating S-glutathionylation of nuclear factor kappa-light-chain-enhancer of activated B cells (NF-κB) or other proinflammatory signaling pathways [138,139,140], although its importance in regulating smoking-induced lung inflammation and injury is less clear [141,142,143]. Additionally, during the progress of chronic pulmonary diseases such as chronic obstructive pulmonary disease (COPD) or asthma, alteration of the redox homeostasis leads to dysregulation of Nrf2 and defective antioxidant signaling [144,145,146].

Considering treatments, among the traditional treatments are inhaled corticosteroids (ICS) [147,148]. These reduce inflammation by modulating NF-кB and AP-1 expression, and are effective preventing oxidative stress [149,150]. In new onset untreated persistent asthma, low-dose inhaled budesonide reduces asthma exacerbations by almost 50% [151], and ICS can reduce the number of airway eosinophils [152,153,154]. A combination of ICS and long-acting β2-agonists (LABAs) (e.g., budesonide and formoterol) significantly reduces asthma exacerbations compared with ICS alone [155]. Alternative treatments are antileukotrienes [156], the anticholinergic tiotropium [157], and environmental control [158].

Nevertheless, even in patients with well controlled asthma, exacerbations occur. Inhaled or nebulized short-acting β2-agonists (SABAs), such as albuterol or levalbuterol, provide rapid relief of symptomatic asthma [159]. Adding the short-acting anticholinergic ipratropium bromide to the inhaled SABA albuterol in severe exacerbations improves lung function and decreases rates of hospitalization [159,160,161]. Moreover, several studies have assessed ICS and oral corticosteroids (OCS) during moderate or severe acute asthma exacerbations. However, the evidence for the efficacy of these agents remains controversial because of significant safety concerns and weak outcomes [148,162,163].

#### 4.2.2. Chronic Obstructive Pulmonary Disease (COPD)

COPD is mainly initiated by inhalation of cigarette smoke, which induces oxidative damage to lung epithelial cells, leading to the development of a chronic persistent inflammatory response from the peripheral airways to the lung parenchyma [164]. Cigarette pollutants, through PRRs such as TLRs and purinergic receptors, and damage-associated molecular patterns (DAMPs) released by apoptotic or necrotic cells, readily initiate pattern recognition [165]. This is followed by the local buildup of chemotactic factors, which attract inflammatory cells to the injured region [166,167,168]. Macrophages, epithelial cells, and infiltrated inflammatory cells release, on the one hand, proteases such as matrix metalloproteinase 9 (MMP9), resulting in elastin degradation and emphysema [169,170]. Thereby the immune system switches to a Th17 response to promote inflammation [171,172] and, on the other hand, also secretes transforming growth factor-β (TGF-β), a pleiotropic cytokine that triggers tissue remodeling and fibrosis [173,174]. Furthermore, the airway smooth muscle produces inflammatory cytokines, proteases, and growth factors, which are essential in the remodeling process and induce structural and functional changes in the airways [175].

ROS directly damage biological molecules and lung extracellular matrix, leading to cell dysfunction or death, and activate NF-κB, which enhances the expression of inflammatory genes such as IL-8 and TNF-α, important in COPD, contributing to reversible airway narrowing [176]. Neutrophils show increased oxide anion (O_2_^−^) levels [177], and lipid peroxidation products such as thiobarbituric acid (TBA), linoleic acid (LA), and F2-isoprostane are also increased [178]. Conversely, the plasma antioxidant capacity is decreased [179]. Oxidative stress contributes to a proteinase–anti-proteinase imbalance, both by inactivating anti-proteinases such as α1-antitrypsin (A1AT) and secretory leukocyte proteinase inhibitor, and by activating MMPs [180,181]. As occurs in asthma, GSH levels are decreased and hold a more oxidized state [182]. In contrast, lung SOD2 levels are increased in response to hyperoxia and inflammatory cytokines [183]. Genetic studies have shown that SOD3 is associated with lung function and the development of COPD [184,185,186]. TRX may be reduced in COPD [187]. Increased levels of extracellular GRX-1 have also been reported in the lung and sputum of asthma and COPD patients [139,188], although its relevance regulating smoking-induced lung inflammation and injury is still unclear [141,142]. Likewise, induction of Nrf2-mediated antioxidant gene expression seems to be flawed [144,189].

Historical treatments for COPD have primarily focused on long-term oxygen therapy and restoring pulmonary function by tackling the underlying inflammation and bronchoconstriction causing air flow obstruction (shortness of breath, cough, chest tightness, and mucus production) [190]. Guidelines recommend the use of long-acting bronchodilators such as LABAs, with or without ICS, or long-acting anticholinergics, and, in severe exacerbations, the addition of phosphodiesterase inhibitors, including roflumilast [191]. Oral corticosteroids are often used in patients that fail to respond to these therapies.

#### 4.2.3. Pulmonary Fibrosis

Pulmonary fibrosis is the outcome of a diverse group of lung insults including toxins, fibers/particles, autoimmune reactions, drugs, and radiation. Nevertheless its primary etiology is unknown and in most cases is referred to as idiopathic pulmonary fibrosis (IPF) [192]. Aberrant epithelial-mesenchymal crosstalk is the main hallmark of IPF, although the immune-inflammatory process plays an important role in all stages of fibrosis. Neutrophil accumulation is a characteristic feature of alveolitis [193,194], leading to persistent injury, tissue remodeling and fibrosis, possibly through the release of proteases such as elastase. Macrophages initially secrete proinflammatory cytokines and later recruit fibroblasts, epithelial and endothelial cells, participating in the reparative environment. M2 macrophages seem to play a key role regulating fibrosis [195]. In fact, the risk for IPF acute exacerbations seems to be reflected by M2 cytokine production levels [196]. Monocytes also play a key role during fibrogenesis; they serve as precursor cells for pro-fibrotic macrophages and fibrocytes [197,198], become highly activated during fibrotic conditions, and their presence correlates directly with the extent of fibrosis in the lungs [199]. Fibrocytes are circulating mesenchymal progenitor cells that contribute to fibroblast activation, extracellular matrix production, and other paracrine functions, leading to tissue remodeling and ongoing fibrosis [200,201]. Furthermore, recent studies have suggested that type 2 innate lymphoid cells (ILC2) communicate with mast cells to release mediators that are able to modulate the disease rather than its pathogenesis [202,203]. Regarding the adaptative immune system, T cells are widely present in active-disease regions and pulmonary tertiary lymphoid organs (TLOs) in patients with IPF [204,205,206]. A Th1/Th2 immune response imbalance has been considered essential in IPF pathogenesis. Certainly, Th2 cytokines promote pro-fibrotic responses, whereas Th1 cytokines (IFNγ and IL-12) may be protective [207,208,209,210,211]. Historically, it has been thought that regulatory T cells (Tregs) had a protective role in IPF because of their anti-inflammatory and anti-fibrocyte accumulation activities. Nevertheless, recent studies have indicated that the role of Tregs differed among the distinctive stages of fibrosis. Indeed, Tregs seem also to secrete pro-fibrotic cytokines [212].

Tilting the balance between oxidant production and antioxidant protection towards the former leads to ROS accumulation, which is promoted by fibrotic stimuli of unidentified origin [213,214]. In fact, oxidants may contribute to the occurrence of pulmonary fibrosis because of their effects on the secretion of cytokines and growth factors such as TGF-β [215,216]. TGF-β induces ROS production by activation of NOX (e.g., NOX4) [217,218,219] or mitochondrial dysfunction. It also reduces the expression of both catalase and SOD2, and thereby decreases natural cellular antioxidant production [220,221,222,223]. Human subjects with IPF have shown increased levels of oxidized proteins [211,224]. As in asthma and COPD, GSH levels are often suppressed and occur in a more oxidized state [225]. Animal studies in mice have demonstrated a protective role of SOD3 in models of interstitial lung disease, including bleomycin-induced pulmonary fibrosis [226,227,228,229], which have been confirmed in patients [230]. TRX expression is also upregulated in pulmonary fibrosis and interstitial lung disease [231,232].

There has been no evidence for an effect of corticosteroid treatment in patients with IPF [233]. In addition, N-acetylcysteine, either alone or with azathioprine and prednisone, offered no significant benefit and was even detrimental [234,235]. In the last years, two anti-fibrotic drugs have been introduced for IPF treatment: pirfenidone and nintedanib [236,237,238], and both decrease the progression of the disease and enhance its evolution. In severe disease, the possibility of lung transplantation should be considered [239]. For the treatment of persistent hacking cough among people with IPF, dimemorphine phosphate, codeine phosphate, prednisone, or thalidomide are indicated [240]. Low doses of morphine improve the sensation of breathlessness and cough in patients with advanced disease. Among non-pharmacological treatments, home oxygen therapy is feasible in patients with IPF and hypoxemia or respiratory rehabilitation [241].

### 4.3. Cystic Fibrosis

**Figure 1:**
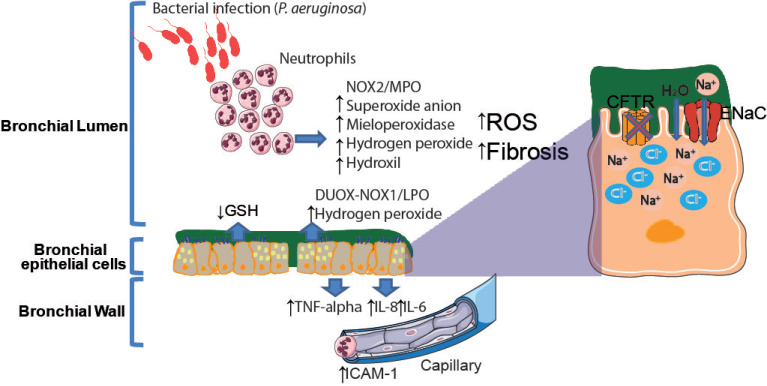
Redox imbalance in the conductive airways of patients affected by cystic fibrosis (CF). Cystic fibrosis transmembrane conductance regulator (CFTR) dysfunction in lungs prevents Cl^−^ secretion and induces Na^+^ hyperabsorption at the airway apical surface, dehydration and impairment of mucociliary clearance. These events favor bacterial infection and prevent its elimination, inducing epithelial cells to secrete proinflammatory cytokines such as IL-8 and IL-6 and TNF-alpha, which attract neutrophils at the site of infection. As a result, a vicious cycle of neutrophilic inflammation and oxidative stress, produced by the release of large amounts of reactive oxygen species (ROS) both from epithelial cells and neutrophils through DUOX and NOX2, respectively, leads to irreversible airway destruction and fibrosis. Furthermore, low glutathione (GSH) levels further increase the oxidative stress. ROS also increase the migration of neutrophils from capillary venules.

Cystic fibrosis (CF), the most frequent recessive hereditary autosomal disease in the Caucasian population, is caused by mutations in the cystic fibrosis transmembrane conductance regulator (*CFTR*) gene. Despite the occurrence of multiorgan complications in CF, respiratory failure secondary to chronic lung infection caused by recurrent bacteria and the subsequent inflammation is the most serious outcome of this condition [242,243]. Thus, abnormalities in the CFTR chloride (Cl^−^) channel at the airway apical surface cause the failure of Cl^−^ secretion and, consequently, sodium (Na^+^) hyperabsorption in epithelial cells, dehydrating the airway surface fluid layer, and impairing mucociliary clearance [244,245,246]. These events support bacterial infection [247,248], which attracts neutrophils (the largest source of ROS) to the site of infection (Figure 1). This infection-inflammation vicious cycle leads to irreversible airway destruction and fibrosis [249]. Although neutrophils, epithelial cells, and their effectors have been most intensely studied, many other cell types, including dendritic cells, T and B lymphocytes, macrophages, and airway smooth muscle cells produce inflammatory mediators and are actively involved in the host inflammatory response in CF [250].

The DUOX/LPO system is engaged by epithelial cells to produce significant quantities of ROS (H_2_O_2_) through two isoforms of NOX expressed in their apical membrane (DUOX 1 and DUOX 2) thought to be related to the CFTR channel [37]. An important function of DUOXs is to support lactoperoxidase (LPO), which is released by caliciform cells of the airways and by the submucosal glands to generate bactericidal hypothiocyanite from thiocyanate and H_2_O_2_ [251]. This system is activated continuously and independently of the presence of an open bacterial infection, unlike NOX/MPO [252]. Multiple studies have shown the antimicrobial function of DUOX/H_2_O_2_ and LPO either against bacteria [253] or viruses [254]. Even mammalian airway epithelial cells might present a novel innate defense mechanism killing bacteria through ROS generation [255]. In CF patients, the level of NO is reduced, and this is directly related to a deterioration of the pulmonary function [256,257]. A sub-optimal antioxidant protection is observed [258]. There are other proteins regulating the H_2_O_2_ levels, such as TRX1, GSTP1, PRDX6 [259], PRDX3 [51], or catalase [260], which are decreased in CF patients. To connect these modifications with CF specific features, a dysfunctional CFTR channel has been found to be associated with a reduced activity of Nrf-2 [261,262].

The presence of a defective CFTR appears to induce a pro-oxidative imbalance in epithelial cells and extracellular fluids of CF airways, which increase the levels of ROS. Constitutive defects on GSH reductase and NOX activities [263,264,265,266] together with intestinal malabsorption of fat-soluble antioxidant vitamins (vitamin E and carotenoids) [267,268] could promote a defective antioxidant protection and exacerbate oxidative stress indices, contributing to the progression of the clinical status in CF patients [269,270,271].

Moreover, ROS activate second messengers through phospholipases A2, C, and D, which induce cytokines and mucin production. Oxidative stress and inflammation can also affect surfactant biophysical activity, leading to early alterations of lung function in patients with CF [272].

Antibiotic treatment has been shown to prevent and control lung infections. These mainly consist of inhaled, nebulized or aerosolized forms of azithromycin, aztreonam and levofloxacin [273,274,275]. Alternatively, ciprofloxacin, cephalexin, amoxicillin, and doxycycline have also been recommended depending on the sensitivity patterns of the bacterial pathogens [276,277]. Colistin is also used [278]. To control airway inflammation, nonsteroidal anti-inflammatory drugs (NSAIDs) [279,280] and cromoglycate (cromolyn) [281] are used. To reduce the viscoelasticity of bronchial mucus secretions and promote its clearance from CF lungs, and to dilate the airways, bronchodilators such as inhaled β-agonists with humidified oxygen, a 3–6% hypertonic saline solution [282,283,284], and dornase alfa [285,286,287,288] are recommended. Furthermore, exercise [289] and chest physiotherapy including oscillating devices for mucus mobilization is being prescribed as well [290].

## 5. Prospective Therapeutic Strategies

### 5.1. ALI/ARDS

The scientific rationale for emerging therapies in ARDS is to pursue fundamental processes and mediators of its complex pathophysiology [291]. There are emerging therapies in Phase 3 trials assessing the potential benefits of corticosteroids such as dexamethasone [292,293] or budesonide/formoterol [294,295]. Moreover, supplementation with vitamin D [296] in a Phase 2 trial has shown a reduction in markers of vascular permeability from lung injury patients following esophagectomy in the post-operative period [297], although another trial using vitamin D to prevent acute respiratory tract infections has been less conclusive [298]. Other emerging therapies in Phase 2 trials include aspirin, which has attracted interest as a repurposed drug for ARDS [299,300], with some clinical studies [301] that show significant reduction in neutrophil infiltration into the alveolar space. Alternatively, different studies using mesenchymal stem cells (MSCs) and multipotent adult progenitor cells (MAPCs) have shown a biological decline in angiopoietin and a concomitantly reduced 28-day mortality, higher ventilator-free days, and higher ICU-free days [302]. Vitamin C acts as ROS scavenger, modulator of inflammatory mediators, and cofactor. In mouse models, ALI prevents the activation of NF-κB, and therefore attenuates the production of proinflammatory cytokines and boosts ion channel and pump expression, enhancing fluid clearance in the alveolar epithelium [303]. The phase 2 CITRIS-ALI trial is presently investigating the usefulness of vitamin C in sepsis-induced ALI [304] but no positive results have been reported as yet [305]. It was found that nebulized liquid heparin increased the number of ventilator-free days [306], and a Phase 2 trial to confirm the findings is awaited [307]. Anti-tissue factor antibodies such as ALT-836, which blocks binding to coagulation factor VIIa, have demonstrated attenuation of sepsis-induced ALI in animal models, and was successfully tested in a Phase 1 trial for ARDS [308]. A Phase 2 trial has been recently completed and disclosure of the results is pending [309]. Dilmapimod, a p38 MAPK inhibitor, has proven useful for reducing the severity of ALI in animal studies, although in human trials it has been unreliable [310,311]. Neutrophil elastase inhibitors such as sivelestat, have been shown to increase the ventilator-free days in ARDS patients with a high extravascular lung water content (>10 mL/kg) as compared with those with low pulmonary edema [312], although contradictory results have also been obtained [313,314]. Ulinastatin (or urinary trypsin inhibitor) is another physiological inhibitor of human neutrophil elastase with positive results in preclinical studies [315]. A meta-analysis of 29 Chinese randomized controlled trials (RCTs) indicated that ulinastatin was effective ameliorating ARDS [316]. Another multi-center Phase 2 RCT is ongoing to assess its safety and efficacy in ARDS [317]. Regarding granulocyte-macrophage colony stimulating factor (GM-CSF), preclinical models have demonstrated that it can limit alveolar epithelial cell injury and promote alveolar macrophage maturation. Nevertheless, a Phase 2 RCT enrolled only two-thirds of its intended number of participants and, although GM-CSF treatment appeared to be safe, it did not decrease ventilator free days or mortality of the ALI/ARDS patients [318]. Anti-CD14 antibodies protected against septic hypotension in animal models of pneumonia [319]. Two Phase 2 trials have been initiated in this regard, the former, in 2007, failed in recruiting people; and the latter is still recruiting [320]. Inhaled prostaglandins, such as epoprostenol and alprostadil, have been suggested to regionally dilate the pulmonary vasculature increasing arterial oxygenation in ARDS. However, a meta-analysis of 25 studies concluded that, although indeed inhaled prostaglandins improved oxygenation in ARDS, they did not improve pulmonary physiology or mortality [321].

#### Preclinical Studies

The focus of preclinical studies has been basically on antioxidants, antiproteases, and signal transduction inhibitors. Histones have strong proinflammatory, cell damaging, and procoagulant activities in the airways. Targeting histones in the lung with neutralizing monoclonal antibodies (mAb) provides high-level protection against the development of ALI [322]. An alternative strategy for ALI-induced sepsis employed a non-anticoagulant heparin derivative that binds to histones and prevents histone-mediated cytotoxicity, mitigating mortality in sepsis mouse models [323]. Because of the variety of chemokines and cytokines, and the overlapping interactions with their receptors, in vivo blockade of these mediators with mAbs would likely not be effective in ALI patients. Nonetheless, the IL-1β receptor antagonist (anakinra), which neutralizes inflammasome-derived IL-1β and has proven effective in rheumatoid arthritis [324] and in complement-dependent collagen-induced arthritis in mice [325], might also improve ARDS outcomes. Among the antioxidants, there are several natural products such as curcumin, ginsenoside, alpinetine, and honokiol [326].

### 5.2. Asthma

Over the last few years, multiple biologics (typically mAbs) have been developed targeting various participants in allergies and asthma, but mainly directed toward the complex type 2 endotype [327,328]. In general, they are anti-inflammatory treatments [329]. The most prevalent biologics are omalizumab (anti-IgE) and mepolizumab (anti-IL-5). IL-5 has become a major target for both asthma and COPD due to the high proportion of patients with airway eosinophilia associated with disease severity [330]. Currently, three biologics, targeting IL-5 or its receptor, have been cleared by the Food and Drug Administration (FDA). Omalizumab was initially approved by the FDA in 2003 and binds to both the high-affinity and low-affinity IgE receptors, preventing free IgE from occupying the surface of mast cells and basophils [331]. It has several disadvantages, i.e., it must be administered by subcutaneous injection [332], it is expensive [333] and, moreover, an unusual form of anaphylaxis [334] and a possible higher rate of cardiac and cerebrovascular events can be ensued by this treatment. Anti-IL-5 (mepolizumab) was approved in late 2014 and receiving patients had decreased eosinophilic inflammation, reduced asthma exacerbations, improved asthma control markers, better quality of life [335,336], and reduced levels of some of the proteins that drive airway remodeling [337]. In another study on moderate persistent asthma, despite high-dose ICS, patients also showed decreased blood and sputum eosinophils but no change in FEV1, symptom scores, or need for rescue inhaler. After stopping anti-IL-5 treatment, eosinophils and asthma symptoms again increased [338]. Reslizumab, another mAb targeting IL- 5, approved in 2016 for patients with eosinophilic asthma, has proven beneficial on moderate-to-severe asthma symptoms, improving lung function and reducing exacerbations as compared with a placebo [339]. Reslizumab also decreased blood, sputum, and airway eosinophils and, more recently, reduced systemic corticosteroid dosing nearly 75% [340,341,342]. Benralizumab, a mAb targeting the IL-5Rα, was approved recently by the FDA [343] with positive results in asthma [344,345,346]. Finally, dupilumab, a mAb approved in 2017 that inhibits the IL- 4R subunit [347], has also shown encouraging results in asthma [348,349]. Regarding relevant future targets pending approval, lebrikizumab and tralokinumab, mAbs that target IL-13 [350,351], have not shown positive effects [352,353]. Tezepelumab (AMG157), a humanized mAb currently in Phase 3 [354] binds thymic stromal lymphopoietin, an epithelial cell-derived cytokine that drives allergic inflammatory responses [355]. Additionally, anti-IL-33 therapies are currently under development [356]. Conversely, non-Th2 inflammation targets are also being studied. IL-6 and IL-17 may promote both Th2 and non-Th2 inflammatory cascades. Brodalumab is a human mAb binding IL-17RA, which inhibits signaling of IL-17 and IL-25, with disappointing results in clinical trials [357]. Thus, this therapy has not been further pursued for asthma or COPD. C-X-C motif chemokine receptor 2 (CXCR2) antagonists such as navarixin (which decrease IL-8 levels) have reduced sputum and blood neutrophils, with no significant change in FEV1 [358], but has progressed to a Phase 2 trial [359]. An antisense oligonucleotide against C-C chemokine receptor 3 (CCR3) (co-administered with an antisense oligonucleotide that targets the c subunit of the IL-3, IL-5, and GM-CSF receptors), named TPI ASM8, has shown some efficacy in phase 2 trials [360]. Imatinib is a tyrosine kinases inhibitor that has shown promising results in a clinical study, reducing airway hyper-responsiveness as compared with a placebo [361]. Among drugs targeting TNF-α, etanercept stands out as a repositioning drug for asthma. A few studies employing etanercept have reported satisfactory results reducing bronchial hyperreactivity [362,363], whereas other studies have informed poor clinical efficacy in terms of lung function improvement and quality of life [364]. Others have shown a small but significant increase in the quality of life without changes on lung function [365].

Among anti-inflammatory treatments, antioxidant treatments stand out [98,366]. Vitamins (E, C, D, and A), carotenoids (α-carotene, β-carotene, β-cryptoxanthin, lutein/zeaxanthin, and lycopene), and food supplements (selenium and zinc) seem to improve the prognosis of the disease [102,367,368]. Asthmatic adults with antioxidant-poor diets have lower forced expiratory volume in the first one second to the forced vital capacity (FEV1/FVC) ratio scores, increased plasma C-reactive protein, and were more likely to exacerbate than those on an antioxidant-rich diet [369]. Indeed, dietary antioxidant supplementation notably improved both symptoms and lung function in exercise induced asthma [370].

#### Preclinical Studies

Thiol-based antioxidants are popular supplements to trigger GSH conversion. N-acetyl cysteine is the most commonly used thiol precursor. Thus, N-acetyl cysteine supplementation remarkably decreased inflammatory cytokines (IL-13, IL-5), neutrophil, and eosinophil numbers in the bronchoalveolar lavage fluid (BALF) from a mouse model [371], which has been associated with excessive bronchoconstriction [372]. Among natural extracts, sakuranetin is a flavonoid that can attenuate airway hyperresponsiveness, while decreasing oxidative stress (8-isoprostane), Th2 proinflammatory cytokines, IgE, and vascular endothelial growth factor levels in the lungs, similar to dexamethasone. Moreover, it can reverse airway remodeling by controlling NF-κB activation [373,374]. Astragalin, another flavonoid, suppresses eosinophil infiltration [375,376]. Resveratrol has been shown to decrease p47phox expression and ROS production, increase SOD levels, and reverse elevated TNF-α and inducible NOS (iNOS) from lung tissue [377,378]. Morin, an active ingredient obtained from Moraceae plants, abolished intracellular ROS and MAPK [86] and attenuated the extensive trafficking of inflammatory cells into BALF from ovalbumin (OVA)-challenged mice and rats [379]. Boerhavia procumbens inhibited oxidative stress pathways, reducing the anti-inflammatory response, and improving lung injury [380]. Esculentoside A and Sonchus asper extract significantly upregulated Nrf-2 expression, SOD activity, and intracellular GSH levels [381,382]. Oral treatment with Capsicum annuum L. methanolic extract remarkably decreased the pathophysiological signs of OVA-induced airway inflammation, reducing ROS levels of BALF in asthmatic mice [383]. Carissa opaca fruit extracts can restore the activities of antioxidant enzymes and lipid peroxidation [384,385]. Furthermore, antioxidant synthetics such as Y-27632, a Rho-kinase inhibitor, controls airway inflammation and responsiveness, the remodeling response, and oxidative stress in guinea pigs [386,387]. Treatment with 1400 W (an iNOS-specific inhibitor), nor-HOHA (an arginase inhibitor), or NaHS (a hydrogen sulfide (H_2_S) donor) decreased pro-contractile 8 isoprostane expression and modulated arginase 2 and iNOS pathways, contributing to reduced NF-κB expression in distal lung tissue. These inhibitors also mitigated eosinophil infiltration and increased tissue resistance and elastance [388,389]. HYDAMTIQ is a promising poly (ADP-ribose) polymerase inhibitor that prevents lung inflammation, airway remodeling and damage in asthma [390]. An angiotensin-I converting enzyme 2 (ACE2) activator, diminazene aceturate, attenuates allergic airway inflammation in a rat asthma model [391]. Diallyl sulfide decreases infiltrated inflammatory cell count and Th2 proinflammatory cytokines in BALF from OVA-challenged mice [392]. Mice treated with S-adenosylmethionine, a potent methyl donor, had decreased amounts of Th2 proinflammatory cytokines and 4-hydroxy-2-nonenal in lung tissues [393]. Pituitary adenylate cyclase-activating polypeptide reverses vanadate-induced airway hyperresponsiveness in rats, mainly through bronchodilator activity and counteraction of proinflammatory and pro-oxidative effects [394].

Furthermore, a multiple targeting approach against oxidizing molecules, PRRs and transcription factor modulators could improve outcomes in asthma. Flavonoids have stronger radical scavenging activity as compared with many other natural antioxidants because of multiple hydroxyl groups present in its chemical structure [395,396,397,398,399]. Ambroxol is a mucoactive agent with anti-inflammatory and antioxidant activities used to increase mucociliary clearance and regulate surfactant levels in the upper respiratory airways [400]. The salicylic acid derivative 5-aminosalicylic acid reduces leukocyte count, the expression of Th2 cytokines, and oxidative stress markers in the BALF from asthmatic mice [401]. Sitagliptin and Cinnarizine also reduce proinflammatory cytokine release and inflammatory infiltration, while restoring GSH and SOD, thus, playing a role attenuating airway inflammation and remodeling through antioxidative stress [402,403]. However, some of the above approaches have failed due to unanticipated side effects. Thus, as yet, no antioxidants have been employed as first-line therapy for asthma.

### 5.3. COPD

Thus far, no anti-IL-5 therapies have been approved for use in COPD. However, two Phase 3 studies using mepolizumab showed improvements in exacerbation frequency from subjects who had an eosinophilic phenotype and a history of COPD exacerbations, despite triple therapy [404]. Nevertheless, other studies have indicated no positive effects [405]. Reslizumab has yet to be formally evaluated in clinical trials for COPD. Conversely, in a Phase 2 trial including COPD patients with eosinophilia, benralizumab treatment did not significantly reduce the annual rate of moderate or severe exacerbations [406,407]. However, significant improvements in FEV1 were observed in the overall study population, and the results of pre-specified subgroup analyses by baseline blood eosinophil count in individuals with benralizumab versus placebo have led to an ongoing Phase 3 trial to evaluate this biologic in COPD [408]. As in asthma, non-Th2 inflammation targets include CXCR2 and CCR3. Regarding corticosteroids, in contrast to asthma, glucocorticoid treatment of established COPD is rather ineffective in reducing chronic airway inflammation and progressive airway obstruction [409]. Current national and international guidelines endorse the use of inhaled long acting bronchodilators, ICSs, and their combination for maintenance treatment of moderate-to-severe stable COPD [410], although adverse effects may arise [411]. In fact, large clinical trials assessing the combination therapy (ICSs + LABAs) in a single inhaler for stable COPD patients have shown a good safety profile, a discreet but statistically significant reduction of severe exacerbations, and improvements of FEV1, quality of life, and respiratory symptoms in these patients [412,413]. Overexpression of histone deacetylase 2 restores glucocorticoid sensitivity in BAL macrophages from COPD patients [414]. Anti-cytokine and anti-chemokine treatments are being exploited in COPD but scarce trials using blocking antibodies against cytokines and chemokines or their receptors have proven successful [409]. Among those showing positive effects, the CXCR2 inhibitor MK-7123 (also known as SCH527123 or navarixin, already described in asthma) could reduce the chemotaxis of neutrophils [409]. MK-7123 treatment resulted in a significant reduction of sputum neutrophils and of sputum and plasma MMP9 and myeloperoxidase levels [415]. Numerous other drugs, including antibodies directed against specific inflammatory mediators such as cytokines (IL-18, IL-22, IL-23, IL-33, TSLP) and growth factors (GM-CSF) are under investigation for COPD.

#### Preclinical Studies

The treatment of respiratory symptoms is the focus of most therapies for COPD, since therapies for comorbidities are ineffective or inexistent. Thus, there is a need for the identification of the mechanisms which relate COPD to its comorbidities, and preclinical models have been developed to solve this [416]. Pharmacological activation of soluble guanylate cyclase, involved in the nitric oxide-cyclic guanosine 3′,5′-monophosphate (NO-cGMP) signaling pathway, has been shown to prevent the development of emphysema and pulmonary vascular remodeling in animal models of COPD [417]. Regarding the cardiovascular effects of COPD, N-acetyl cysteine or the proteasome inhibitor bortezomib attenuated muscle mass loss and wasting [418]. The inflammation is also very important and airway epithelial cells, oxidative damage, epithelial-mesenchymal transition, and airway remodeling have also been considered to be potential therapeutic targets [419]. The mesenchyme plays a key role in COPD, and bromodomain-containing protein (BRD) along with NF-κB, contribute to mesenchymal transition. Selective inhibitors of the epigenetic regulator BRD4, specifically targeting TLR3-induced airway inflammation, have been developed through structure-based drug design [420,421]. The aim is to decrease the levels of neutrophilic inflammation in the airways and prevent the alteration of the epithelial cell state, reducing myofibroblast growth in infection exposures. MSCs may also be used as a therapeutic strategy for the treatment of COPD, reducing inflammation, antimicrobial actions, and promoting lung epithelial and endothelial repair [422].

### 5.4. Idiopathic Pulmonary Fibrosis (IPF)

After several disappointing years of promising therapies that moved into clinical trials but failed to demonstrate efficacy in IPF [423], the anti-fibrotic drugs pirfenidone and nintedanib have been associated with significantly slower respiratory deterioration and perhaps prolonged survival [236,237,424], although with heterogenous responses and side effects. The understanding of the complex pathogenesis of IPF continues to increase [425,426]. Sustained alveolar epithelial cell injury and abnormal repair are increasingly recognized as the core mediators of the fibrotic process, with a relevant involvement of environmental triggers. The activation of multiple pathways related to maladaptive repair, involving fibroblast migration, proliferation, and extracellular matrix deposition has revealed a variety of prospective molecular targets of novel therapeutic agents currently being tested in early phase clinical trials. Pentraxin-2 (PTX-2) is a circulating protein that binds to monocytes, promoting epithelial healing and resolution of fibrosis. Thus, a recombinant human PTX-2 (serum amyloid P) analogue (PRM-151) has been shown to inhibit monocyte to fibrocyte differentiation and ameliorate fibrosis in a bleomycin-induced animal model of fibrosis [427,428]. A Phase 1 trial showed a non-significant but improving effect of PRM-151 on FVC and six-min walking distance (6MWD) during the treatment [429]. Further Phase 2 studies have demonstrated a significant reduction in pulmonary function deterioration and stability in 6MWD over 24 weeks as compared with a placebo, although with relevant adverse events [430,431]. The launch of a Phase 3 trial for PRM-151 in IPF has been announced, using FVC as a primary end point and 6MWD as a key secondary end point. Among the anti-connective tissue growth factor antibodies, the antagonist pamrevlumab (FG-3019) in the PRAISE study [432] was established to have a significant effect preventing lung function decline of 160 IPF patients, yet full peer-reviewed data are still awaited [433]. The re-initiation of Phase 3 trials has just been announced. PBI-4050 is a synthetic analogue of a medium-chain fatty acid acting through G protein-coupled receptors and showing anti-fibrotic activities such as inhibition of epithelial–mesenchymal transition and fibrocyte/fibroblast recruitment, migration, proliferation and differentiation, among others [434]. A Phase 2 trial has shown no safety concerns [435]. While there was slowing or stability in FVC, a statistically significant decrease was observed only combining PBI-4050 and pirfenidone but not PBI-4050 and nintedanib, implying a possible drug–drug interaction. Additional studies of PBI-4050, either alone or in combination with nintedanib, are currently being considered. In a Phase 2a study [436], GLPG1690, an oral selective inhibitor of autotaxin (an enzyme increased in IPF and involved in cell apoptosis and endothelial cell damage) was analyzed and was well tolerated by IPF patients, with a good safety profile. Moreover, as secondary end points, preliminary efficacy analyses demonstrated target engagement and encouraging results towards halting FVC decline [437]. International Phase 3 trials to assess the efficacy of GLPG1690 in IPF are ongoing [438]. Leukotrienes are also increased in IPF [439,440]. Thus, additional ongoing trials include leukotriene antagonists such as tipelukast, currently explored in a Phase 2 trial [441]. Among protein kinase inhibitors, a recent Phase 1 study showed proper safety and tolerability of a selective protein kinase inhibitor of the Rho-associated coiled-coil containing protein kinase 2 (ROCK2). The trial is currently in Phase 2 [442,443]. Moreover, a current Phase 2 [444] trial is evaluating CC-90001, a second-generation Jun N-terminal kinase (JNK) inhibitor after a first-generation JNK inhibitor (CC-930) showed a dose-dependent trend of reduction in MMP7 and surfactant protein D (SP-D) biomarker plasma levels [445]. Regarding anti-integrin antibodies, a partial inhibition of integrin αvβ6 in rodents blocked the development of pulmonary fibrosis processes without aggravating the inflammatory response [446]. The safety and tolerability of a humanized monoclonal antibody (BG00011) against this integrin has been analyzed in a Phase 2 trial [447]. The study has been completed recently, although its outcome is still pending. Phosphatidylinositol 3-kinase/Protein kinase B (PI3K/Akt) pathway inhibitors may be associated with halting fibrosing processes [448], as suggested in a Phase 1 trial [449,450] and evidenced in another recent study using omipalisib [451]. Sirolimus is currently under examination in a Phase 2 trial [452]. The B lymphocyte antigen CD20 is targeted by rituximab, which is currently being assessed in an IPF Phase 2 study [453]. Furthermore, a Phase 2 trial examined combined plasma exchange, rituximab, and steroids [454]. While peer-reviewed results are pending, a pilot trial stated good outcomes regarding autoantibody reduction for acute IPF exacerbations [455]. A Phase 3 trial testing the antibiotic combination co-trimoxazole (trimethoprim and sulfamethoxazole) is currently operating [456]. Finally, other anti-inflammatory drugs are likewise in clinical research for IPF, i.e., lebrikizumab [457], tralokinumab [458], and azithromycin [459].

#### Preclinical Studies

Administration of the senolytic drug quercetin attenuates the proinflammatory phenotype of bleomycin-induced senescence in fibroblasts [460]. Moreover, the anti-fibrotic activity of quercetin was superior to that of vitamin E in bleomycin-induced pulmonary fibrosis in rats [461]. When given in combination, the tyrosine kinase inhibitor dasatinib and quercetin depleted senescent cells and improved lung function and fibrotic markers following bleomycin injury [462,463,464,465]. Different preclinical and clinical trials testing the senolytic cocktail dasatinib plus quercetin has shown both positive outcomes [466] and negative ones [467,468]. Other senolytics with positive preclinical studies are curcumin, fisetin and fisetin-loaded mesoporous carbon nanoparticles [469], and navitoclax [470]. Therefore, the promising expectations generated in preclinical studies supports proof-of-principle clinical trials with senolytic agents for IPF treatment [471]. Conversely, it has also been suggested that senolytic drugs could be detrimental to IPF patients [460].

### 5.5. Cystic Fibrosis (CF)

To address the most prevalent causal defects in the CFTR Cl– channel leading to CF, two biomolecular modulators are needed, i.e., CFTR correctors, to increase the amount of properly folded mutant CFTR protein at the plasma membrane, and CFTR potentiators, to allow effective gating (channel opening and closing) of the abnormal CFTR [472,473,474]. Nevertheless, a more thorough division might also include stabilizers, read-through agents, and amplifiers [475]. Either alone or combined, these modulators tend to restore transepithelial Cl^−^ transport to CF airway epithelia expressing CFTR mutations such as the most prevalent F508del, improving hydration and restoring mucociliary clearance [476,477]. Four drugs have been recently approved by the FDA for that purpose [474], the potentiator Ivacaftor (VX-770) for individuals with CF holding a G551D CFTR mutation, and the following three correctors: Lumacaftor (VX-809), developed to increase the amount of F508del CFTR that reaches the cell surface [478,479], Tezacaftor (VX-661), and Elexacaftor (VX-445). Furthermore, their combinations are also being assayed, i.e., Orkambi (lumacaftor/ivacaftor) for patients homozygous for F508del CFTR [480], Symdeko (tezacaftor/ivacaftor), and Trikafta (elexacaftor/tezacaftor/ivacaftor). Most recently, there has been an explosion of novel modulators [481] and others are under investigation including ELX-02, Posenacaftor (PTI-801), Galicaftor (ABBV-2222), ABBV-3221, FDL169, Deutivacaftor (VX-561), ABBC-974 (GLPG-1837), and Nesolicaftor (PTI-428) [482,483,484,485,486,487,488,489,490,491], among others.

Despite a thorough knowledge of the undergoing inflammatory process in CF, there are relatively few anti-inflammatory drugs in clinical use [492]. Corticosteroids were shown to confer some benefit but their long-term use is associated with unacceptable side effects [493,494,495]. The non-steroidal anti-inflammatory agent ibuprofen has also demonstrated benefits [279,280]. Particularly in younger patients, it has been associated with an increased survival rate [496,497], but it requires a strict dose control and has associated renal and gastrointestinal side effects [279]. A large Phase 2 RCT of the leukotriene B4 (LTB4) receptor antagonist, BIIL 284 BS (amelubant), surprisingly demonstrated an excess of pulmonary exacerbations as compared with a placebo [498]. Conversely, CTX-4430 decreases the production of LTB4, an inflammatory mediator elevated in CF [499] and is presently undergoing a Phase 2 trial [500]. Andecaliximab, an antibody against MMP9, is undergoing a Phase 2b trial [501] but the baseline FEV1 required for this drug limits its use in very severe CF and this trial has been discontinued. Another compound in Phase 1 is POL6014, a synthetic neutrophil elastase blocker [502]. Other anti-inflammatory compounds under clinical development are α-1 anti-trypsin [503], the elastase inhibitor AZD9668 [504], and JBT-101 (ajulemic acid, or Lenabasum), an oral selective cannabinoid receptor type 2 (CB2) agonist that decreases neutrophilic inflammation inhibiting LTB4 and promotes resolution of inflammation through modulation of arachidonic acid metabolism [505]. A Phase 2, double-blind, placebo-controlled study, in adult CF patients, demonstrated decreased levels of several sputum inflammatory markers and reduced exacerbations in response to JBT-101, with no serious adverse effects reported [506,507]. A Phase 2b study is underway. Indeed, CB2 activation has shown anti-inflammatory effects including stimulating lipoxin A4 (LXA4) synthesis, decreasing proinflammatory cytokine secretion, and neutrophil trafficking to the lung [508,509].

Anti-proteases have been under investigation in CF since 1990. For example, the already described α1-antitrypsin suppressed inflammatory markers including free neutrophil elastase, proinflammatory cytokines, and neutrophils [510,511]. Other neutrophil elastase inhibitors include recombinant secretory leukocyte protease inhibitor (rSLPI) and the small-molecule drug EPI-hNE4 (depelstat) [512]. Among other inflammatory therapies, hydroxychloroquine, a dihydrofolate reductase inhibitor that increases intracellular pH, was negatively evaluated in a small 28-day study in CF [513]. A CF clinical trial regarding SB-656933, a CXCR2 antagonist, concluded that this molecule might modulate airway inflammation [514]. Conversely to refractory asthma, few CF studies have considered the use of chemotherapeutics. Low dose of the immunosuppressant cyclosporin A diminished the need for systemic corticosteroids in one small case series. In a pilot study, methotrexate increased FEV1 and decreased total serum immunoglobulins in five CF patients after one year of treatment [515], showing tolerable adverse effects. IL-8 decoys are used as an anti-inflammatory anti-neutrophil elastase strategy [516,517]. Other novel anti-inflammatory compounds under review include the already mentioned lipoxins and resolvins. Arachidonic acid-derived lipoxins such as LXA4 attenuate neutrophil chemotaxis, respiratory burst, IL-8 production, and accelerate apoptosis [518,519,520]. Because of low LXA4 levels in CF airways, stable LXA4 agonists have been developed as prospective therapeutics. Decosahexanoic acid- and omega-3 eicosapentanoic acid-derived resolvins D1 and E1 also mitigate inflammation, preventing chemotaxis and promoting clearance of apoptotic neutrophils [521,522,523,524]. Analogously to LXA4, resolvins stimulate a cytoprotective effect on airway epithelial cells [525,526]. Retinoids foster extracellular matrix homeostasis. Recent Phase 1b studies involving LAU-7b, an oral solid-dosage form of the retinoid fenretinide, showed safety and tolerability in adult CF patients, encouraging progression to Phase 2 trials [527].

Antioxidant therapies have not been yet settled in clinical practice [528]. In fact, despite the commercial development of many natural antioxidants as dietary supplements, there is no sound clinical trial evidence of their effectiveness in any clinical condition [529] with the exception of GSH (administered either orally or by inhalation) [530,531,532] with some drawbacks [533]. Though not quite clear [534], high doses of β-carotene appear to improve lung function and decrease oxidative stress in some cases [535]. The application of deferiprone (L1) as an iron chelating drug/pharmaceutical antioxidant is under way. Its use is being considered as a main, alternative, or adjuvant therapy in many diseases involving oxidative damage [536,537]. N-acetyl cysteine, initially developed as a mucolytic, is being repurposed as an antioxidant [538], inhibiting H_2_O_2_ and increasing GSH [531]. Of significance is the malabsorption of fat-soluble antioxidants in CF patients such as tocopherols, carotenoids, and coenzyme Q10 (Co-Q10), and that of essential fatty acids. Vitamin E might become a good supplementation to overcome this deficiency [539,540,541,542,543], along with carotenoids [544] and ascorbic acid (vitamin C) as nutritional supplements. Multivitamin supplements with high bioavailability containing Co-Q10 would also be a good alternative [545,546]. One recent study regarding multivitamin supplements showed a decrease in circulating inflammatory markers and a decrease in pulmonary exacerbations [547]. Alternatively, several hydro soluble antioxidants, oligoelements, and enzymatic antioxidants such as Vitamin C, selenium and selenium-dependent peroxidases [548,549,550], zinc, and copper [551] have yielded promising results awaiting further clinical trials. A randomized double-blind placebo-controlled trial has examined the outcome of short-term melatonin administration (3 mg for three weeks) on sleep and oxidative stress markers in CF [552]. Accordingly, with the expected activity synchronizing the sleep-wake cycle and its antioxidant properties, treatment with this hormonal substance reduced nitrite levels in exhaled breath condensate and improved sleep indices.

#### Preclinical Studies

Anti-inflammatory cytokines and antibodies to proinflammatory cytokines may show efficacy in CF. IL-10 possesses anti-inflammatory properties. Interferon-γ1b, another cytokine with immunomodulatory activities, did not improve pulmonary function nor alter bacterial burden or inflammatory markers in the sputum in a multicenter clinical trial, despite its ability to restore macrophage activation and antimicrobial, antiproliferative, and anti-fibrotic functions in CF cell models [553]. To inhibit specific proinflammatory mediators, antibodies to intercellular adhesion molecule 1 (ICAM-1) and IL-8 have been evaluated in preclinical studies [554], although they never progressed to clinical trials. Anti-IL-17 antibodies reduced airway neutrophilia in mice exposed to LPS [555]. Because of the similarities between CF airway inflammation and hyperinflammatory conditions such as rheumatoid arthritis or psoriasis, for which clinical trials using anti-IL-17 antibodies have been completed, targeting IL-17 could also be of therapeutic value for CF treatment.

Among intracellular signaling modulators, ibuprofen (previously described), and IL-10 inhibit NF-κB activation. NF-κB activity inhibition also occurs through upregulation of peroxisome proliferator activating receptor (PPAR-γ) using PPAR-γ agonists such as thiazolidinediones (glitazones) [556,557,558]. Troglitazone and ciglitazone activate PPAR-γ in primary CF airway epithelial cells and reduce the production of proinflammatory mediators in response to *P. aeruginosa* [559]. Statins have anti-inflammatory effects, including the ability to inhibit neutrophil migration, decrease proinflammatory cytokine production, and increase transcriptional activation of PPAR [560,561,562].

Agents that augment endogenous NO production, exhibiting anti-inflammatory activity, have also been examined. In a rodent model of chronic airway infection, L-arginine, which increases NO production, was associated with reduced tissue damage, decreased neutrophil recruitment, and reduced IL-1β [563]. Synthetic triterpenoids are small-molecule derivatives of naturally occurring compounds holding cytoprotective functions that increase antioxidant Nrf2 activity, and therefore could also be considered for clinical trials.

In addition, consumption of long chain fatty acids (omega-3 supplements) seems not to have a clear anti-inflammatory effect and, consequently, a positive benefit on CF disease severity [564].

Among emerging antioxidant and anti-inflammatory approaches for CF, targeting NF-κB with natural compounds such as resveratrol and plant extracts is highlighted. Resveratrol (3,5,4′ trihydroxystilbene, “E” form) is one of the most investigated natural antioxidants with a purported activity as NF-κB inhibitor. The antioxidant activity of resveratrol has been reported in several studies and also occurs in lung tissues, suggesting that resveratrol has potential as a therapeutic agent in respiratory diseases [260,565,566]. The drug Meveol^®^ is a complex of lactoferrin and the anion hypothiocyanite (OSCN^−^), with a proposed in vivo antimicrobial activity [567] to be evaluated in clinical trials as aerosol treatment for lung infections in CF patients. A role for this drug in the control of H_2_O_2_ levels in the airway surface liquid remains to be investigated. In addition, preclinical studies [568] have shown novel anti-inflammatory effects and antimicrobial potential of the anion thiocyanate (SCN^−^) in lung infection, which decreases BALF chemokine keratinocyte chemoattractant (KC) (analog to human IL-8), IL-1β, TNF-α, and airway neutrophil infiltrate concurrent with the infectious stimulus, and enhances bacterial clearance in both wild-type and transgenic mice overexpressing the β subunit of the epithelial sodium channel Scnn1 (βENaC). Therapeutic potential of selenium-derived compounds may derive from their significant activity as inducers of phase II enzymes such as quinone reductase and GSH-S-transferase [569], increasing the capacity to metabolize/detoxify endobiotics generated during oxidative stress and inflammation [570].

## 6. Concluding Remarks

**Figure 2:**
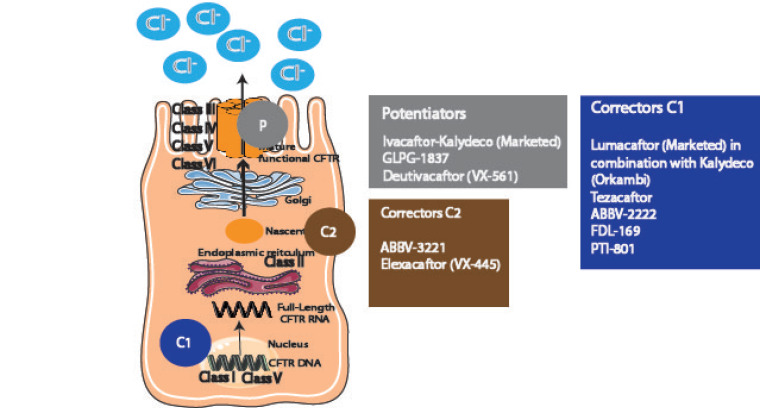
Different CFTR modulators and their targets. Abnormal CFTR protein biosynthesis/function according to the following different classes of CFTR gene mutations: Class I mutants involve no protein synthesis. Premature stop codons, frameshifts, or deletions preclude translation of full-length CFTR; Class II mutants, among them the most common mutation, F508del, have impaired trafficking due to incorrect folding; Class III mutants have defective channel gating; Class IV mutants hold reduced function, such as reduced chloride (Cl^−^) conductance; Class V mutants allow a reduced CFTR cannel number or maturation as a result of amino acid substitution or alternative splicing; and Class VI mutants support a less stable protein, since the recycling CFTR channel is sent for lysosome degradation. These phenotypes can be corrected by rationally designed drugs or drug combinations termed correctors (C1, targeted to class I and class V mutations and C2, targeted to class II mutations), and potentiators (P, targeted to class III–VI mutations).

Because inhaled oxygen along with environmental pollutants, pathogens, and allergens, which have intrinsic oxidative potential, dissolve in the respiratory epithelial lining fluid, oxidative stress and inflammation take center stage in the wide spectrum of respiratory pathologies, a major source of disability and death following cardiovascular diseases. Thus, a timely diagnosis, treatment, as well as the management of pulmonary diseases is important. In that sense, preventive measures such as enhancement of antioxidant defenses or corrective actions to mitigate abnormal immune-inflammatory responses, whether efficacious, could provide more options for patients and clinicians. Nevertheless, the last two decades have witnessed no major breakthroughs regarding medications for most of the respiratory diseases (Table 1). On the one hand, traditional adjuvant therapies such as mechanical lung ventilation for ALI/ARDS or chest physiotherapy and antibiotic therapy in CF improve patient survival slightly. On the other hand, the use of corticosteroids has become commonplace as anti-inflammatory treatment, although with limited efficacy and relevant side effects. Thus, novel drugs or drug combinations targeting relevant players in the disease process, such as neutrophil elastase inhibitors for ALI/ARDS and CF, antibodies against cytokines, chemokines or its receptors for asthma, COPD and CF, anti-fibrotic drugs for IPF, and, ultimately, the recently approved CF therapies targeting CFTR mutations (Figure 2) might represent a step forward to increase specificity and reduce adverse events. Moreover, new cutting-edge technologies, including single-cell next-generation sequencing, genome-wide in silico or phenotypic screenings to identify novel drug targets, and stem cell therapeutics should yield more efficacious and safe drugs to approach personalized treatments for a variety of airway dysfunctions. In fact, by gaining a better understanding of lung pathophysiology at the molecular level using new emerging technologies, the achievement of better outcomes in patients with respiratory pathologies will become a reality.

## Figures and Tables

**Table 1:** Most relevant drugs according to respiratory disease type.

Disease	Drug	Target	Biologic Function	Trial	Reference
ALI/ARDS	Corticosteroids (dexamethasone, budesonide and formoterol)	Corticosteroid receptors	Reduce the signs and symptoms of inflammatory conditions	Phase 3	[292,293,294,295]
ALI/ARDS	Aspirin	Cox-1 and Cox-2	Significant reduction in neutrophil infiltration into the alveolar space	Phase 2	[299,300,301]
ALI/ARDS	MSCs and MPAs	-	Reduction in angiopoietin decreased 28-day mortality, higher ventilator-free days and higher ICU-free days	Phase 2	[302]
ALI/ARDS	ALT-836	Tissue factor (TF) or TF-factor VIIa	Blocks binding to coagulation factor VIIa and attenuation of sepsis-induced ALI	Phase 2	[308,309]
ALI/ARDS	Dilmapimod	p38 MAPK Inhibitor	Reduces severity of ALI	Phase 2	[310,311]
ALI/ARDS	Ulinastin	Physiological human inhibitor of neutrophil elastase	Effective in ameliorating ARDS	Phase 2	[315,316,317]
ALI/ARDS	Anti-CD14 antibodies	Amti-CD-14	Antibodies which protect against septic hypotension	Phase 2	[319,320]
Asthma and COPD	Omalizumab	Anti-IgE	Binds to free human IgE, forming small-size immune complexes, blocking its interaction with the high-affinity IgE receptor and preventing its contact with mast cells and basophils	Approved	[331,332,333,334]
Asthma and COPD	Mepolizumab	Anti-IL-5	Decreases eosinophils in blood and sputum; fewer asthma exacerbations, better asthma control, improved quality of life, and reduced proteins involved in airway remodeling	Approved for asthma; Phase 2 for COPD	Asthma: [335,336,337,338]/COPD: [404,405]
Asthma and COPD	Reslizumab	Anti-IL-5R	Decreases blood, sputum, and airway eosinophils, reduces asthma exacerbations, improves lung function, and reduces systemic corticosteroid dosing by as much as 75%	Approved	[339,340]
Asthma and COPD	Benralizumab	Anti-IL-5	Positive results in asthma. Decrease airway eosinophilia	Approved	Asthma: [343,344,345,346]/COPD: [406,407,408]
Asthma	Depilumab	Anti-IL-4R	Positive results in asthma. Decrease airway eosinophilia	Approved	[347,348,349]
Asthma and COPD	Tezepelumab	Humanized monoclonal antibody	Binds thymic stromal lymphopoietin, an epithelial-cell–derived cytokine that drives allergic inflammatory responses	Phase 3	[354,355]
Asthma and COPD	Navarixin	CXCR2 antagonist	Reduces sputum and blood neutrophils; no significant change in FEV1	Phase 2	Asthma: [358,359]/COPD: [409]
Asthma	Etanercept	TNF-α	Reduces bronchial hyperreactivity; small but significant increase in quality of life	Clinical	[362,363,364,365]
COPD	ICS + LABA	Corticosteroid receptors + β-adrenergic receptors	Significant reduction in the number of severe exacerbations and improvement in FEV1, quality of life, and respiratory symptoms in stable COPD patients	Approved	[412,413]
IPF	Pirfenidone and nintedanib	TGF-β and angiokinase	Significant reduction of respiratory deterioration in IPF and, perhaps, prolonged survival	Phase 3	[236,237,424]
IPF	PRM-151	Protein that binds to monocytes promoting epithelial healing and resolution of fibrosis	Ameliorates fibrosis in a bleomycin- and TGF-β-overexpressing animal model of fibrosis	Phase 2, heading for phase 3	[427,429,430,431]
IPF	Pamrevlumab	CTGF	Reduction of lung function decline	Phase 2, heading for Phase 3	[433]
IPF	PBI4050	Analogue of a medium-chain fatty acid. Activates the GPR40 receptor, while it suppresses GPR84 activity,	Inhibition of endoplasmic reticulum stress and ROS production, epithelial–mesenchymal transition and fibrocyte/fibroblast recruitment, migration, proliferation, and differentiation	Phase 2	[434,435]
IPF	GLPG1690	Autotaxin	Selective autotaxin inhibitor. Enzyme increased in IPF and involved in cell apoptosis and endothelial cell damage, and LPA inhibitor	Phase 3	[436,437,438]
IPF	Tipelukast	Leukotriene antagonists	Downregulation of genes that promote fibrosis, such as LOXL2, collagen type 1, and TIMP-1; and genes responsible for promoting inflammation like CCR2 and MCP-1.	Phase 2	[441]
IPF	KD025	Selective ROCK2 inhibitor	Downregulates the ability of T cells to secrete IL-21 and IL-17 in response to T-cell receptor stimulation in vitro; restores disrupted immune homeostasis	Phase 2	[442,443]
IPF	CC-90001	Second-generation JNK inhibitor	Reduces the development of fibrosis, as evidenced by a 48% reduction in collagen and a 53% reduction in α-smooth muscle actin	Phase 2	[444]
IPF	BG00011	Humanized monoclonal antibody targeting the alpha-v beta-6 (αvβ6) integrin receptor	TGF-β suppression as evidenced by reduction in pSMAD2 signaling and TGF-β dependent gene expression in bronchoalveolar lavage (BAL) cells; preclinical models have shown maximal fibrosis inhibition correlating with 70% pSMAD reduction.	Phase 2	[447]
IPF	Omipalisib	PI3K/Akt pathway inhibitor	Halts fibrosing processes	Phase 1	[451]
IPF	Sirolimus	mTOR	Reduces the number of circulating fibrocytes	Phase 2	[452]
IPF	Rituximab	CD20 surface molecule of B lymphocytes	Reduction of autoantibodies, a favorable safety profile and, possibly, stabilization of lung function.	Phase 2	[453,454,455]
IPF	Cotrimoxazole	Antibiotic	Antibacterial drug	Phase 3	[456]
CF	Lumacaftor	CFTR corrector C1	Increases the amount of F508del-CFTR that reaches the cell surface	Approved	[478,479]
CF	Ivacaftor	CFTR potentiator	CF patients possessing a G551D CFTR mutation	Approved	[571]
CF	Orkambi	CFTR corrector (C1)	For patients homozygous for F508del-CFTR; increases the amount of F508del-CFTR that reaches the cell surface	Approved	[480]
CF	Ibuprofen	Cox-1 and Cox-2	Slows the progression of lung disease in children with CF	Approved	[279,280,496,497]
CF	Amelubant	LTB4 receptor antagonist	Eicosanoid modulator; anti-inflammatory activity	Phase 2	[498]
CF	POL6014	Neutrophil elastase function blocker	Clear inhibition of neutrophil elastase in the sputum of subjects with CF after single dosing	Phase 1	[502]
CF	CTX-4430	Leukotriene A4 hydrolase (LTA4H) inhibitor. Decreases the production of LTB4	LTA4H and LTB4 are strongly associated with the development of many conditions involving inflammation, including CF	Phase 2	[499,500]
CF	JBT-101	Selective CB2 agonist. Decreases neutrophilic inflammation by inhibiting LTB4 and promotes resolution of inflammation by modulation of arachidonic acid metabolism	Reduction in some sputum inflammatory markers; reduction of exacerbations in response to lenabasum, with no serious adverse effects reported	Phase 2 heading for Phase 2b	[505,507]
CF	Thiazolidinediones (glitazones)	Inhibition of NF-κB activity through upregulation of peroxisome proliferator activating receptor (PPAR)	Reduce systemic inflammation in polymicrobial sepsis by modulation of signal transduction pathways	Approved	[556,557,558]
CF	Troglitazone and ciglitazone	PPAR activators	Reduce production of proinflammatory mediators in response to *P. aeruginosa*	Approved	[559]
CF	α1-antitrypsin	Serine protease inhibitor	Suppresses inflammatory markers, including free neutrophil elastase, proinflammatory cytokines and neutrophils	Phase 2	[503]
CF	SB-656933	CXCR2 antagonist	Promising modulator of airway inflammation	Phase 2	[514]
CF	LAU-7B	Retinoids	Promotes extracellular matrix homeostasis; safe and well tolerated.	Phase 1b	[527]
CF	Lenabasum	Cannabinoid receptor type 2 (CB2)	CB2 is found primarily on the surfaces of activated immune cells; upon binding to the CB2 receptors, lenabasum triggers the production of proinflammatory mediators, reducing inflammation; reduces the number of inflammatory cells and inflammatory mediators found in the sputum; FEV1 was stable throughout the study for both lenabasum and placebo groups.	Phase 2, heading for 2b	[506]
CF	GSH	Endogenous antioxidant	Improves lung function and decreases oxidative stress	Phase 2	[530,531,532,533]
CF	β-carotene	Natural antioxidant	Improves lung function and decreases oxidative stress	Phase 1	[534,535]
CF	Deferiprone (L1)	Chelating drug/pharmaceutical antioxidant	Used as a main, alternative or adjuvant therapy in many pathological conditions	-	[536,537]
CF	N-acetyl cysteine	Antioxidant	Inhibits H_2_O_2_ and increases GSH	Phase 2b	[531,538]
